# The Cost-Effectiveness of Collagenase Injection Versus Limited Fasciectomy for Moderate Dupuytren’s Contracture: An Economic Evaluation of the Dupuytren’s Interventions Surgery Versus Collagenase Trial and a Decision Analytical Model

**DOI:** 10.1016/j.jval.2025.07.030

**Published:** 2026-01

**Authors:** Qi Wu, Catherine Arundel, Charlie Welch, Puvanendran Tharmanathan, Nick Johnson, Belen Corbacho, Joseph J. Dias, DISC Trial Team, Maria Armaou, Christopher Bainbridge, John Cooke, Michael Craigen, Lydia Flett, Samantha Brady, Catherine Hewitt, Sophie James, Judy Jones, Ada Keding, Catherine Knowlson, Paul Leighton, David Torgerson, David Warwick, Michelle Watson

**Affiliations:** 1 York Trials Unit, Department of Health Sciences, University of York, York, England, UK; 2 University Hospitals of Derby and Burton NHS Trust, Derby, England, UK; 3 University Hospitals of Leicester NHS Trust, Leicester, England, UK

**Keywords:** collagenase clostridium histolyticum, cost-effectiveness, Dupuytren’s contracture, limited fasciectomy, Markov model

## Abstract

**Objectives:**

To compare the cost-effectiveness of collagenase injection (collagenase) and limited fasciectomy (LF) surgery in treating moderate Dupuytren’s contracture (DC) in the United Kingdom over different time horizons.

**Methods:**

An incremental cost-effectiveness analysis was conducted alongside a multicenter, pragmatic, parallel randomized controlled trial (Dupuytren’s Interventions: Surgery versus Collagenase trial), to determine the short-term cost-effectiveness of collagenase compared with LF. A Markov decision analytic model was developed to assess long-term cost-effectiveness.

**Results:**

Collagenase was associated with significantly lower cost and insignificantly lower quality-adjusted life-year (QALY) gain compared with LF at 1 year. The probability of collagenase being cost-effective was more than 99% at willingness-to-pay thresholds of £20 000 to £30 000 per QALY. At 2 years, collagenase was both significantly less costly and less effective compared with LF, and LF became cost-effective above a threshold of £25 488. There was a high level of uncertainty surrounding the 2-year results. Over a lifetime horizon, collagenase generated a cost saving of £2968 per patient but was associated with a mean QALY loss of −0.484. The probability of collagenase being cost-effective dropped to 22% and 16% at £20 000 to £30 000 per QALY, respectively.

**Conclusions:**

Collagenase was less costly and less effective than LF in treating Dupuytren’s contracture. The cost-effectiveness of collagenase compared with LF was time dependent. Collagenase was highly cost-effective 1-year after treatment; however, the probability of collagenase being cost-effective declined over time. The Markov model suggested that LF is more cost-effective over a lifetime horizon. These findings emphasize the importance of longer follow-up when comparing surgical and nonsurgical interventions to fully capture overall costs and benefits.

## Highlights

• Existing evidence on the cost-effectiveness of treatments for Dupuytren’s contracture is inconsistent. This article addresses this gap by using data from a large randomized controlled trial (Dupuytren’s Interventions: Surgery versus Collagenase trial) to conduct a comprehensive economic evaluation comparing the cost-effectiveness of collagenase injection with limited fasciectomy (LF) surgery.
• Collagenase was less costly and less effective than LF and highly cost-effective at 1 year. However, the probability that collagenase is cost-effective declined over time. Over a lifetime horizon, the Markov model indicated that LF is the cost-effective option.
• The time-dependent results suggest the need for longer trial follow-ups when comparing surgical with nonsurgical interventions to accurately capture their overall costs and benefits.

## Introduction

Dupuytren’s contracture (DC) is caused by a progressive fibro-proliferative disease affecting the palmar and digital fascia of the hand. It causes flexion deformities that impair hand function and, consequently, quality of life.^1^^,^^2^ The disease is a genetic disorder, and its prevalence ranges between 0.6% and 31.6% in Western countries, with higher rates observed in men, aged more than 50, and those of Northern European descent.^3^ Currently, there is no known cure for the disease, and treatments for the resulting contracture cost the National Health System (NHS) more than £60 million yearly.^4^^,^^5^

Limited fasciectomy (LF) surgery, during which the thickened and contracted parts of the fascia are removed, is the most frequently used method for correction of DC in Europe.^6^ Alternatively, pharmacological interventions, such as collagenase clostridium histolyticum injection (collagenase), offer a simpler procedure and potentially faster recovery.^7^, ^8^, ^9^ Collagenase is an enzyme that degrades collagen in the thickened and contracted tissue in DC.^7^ This enzyme is directly administered into the cord, and after a few days, manipulation snaps the cord and straightens the contracted joint. Recent systematic reviews have noted insufficient evidence for the cost-effectiveness of LF compared with collagenase, with heterogeneity leading to inconsistent conclusions.^10^^,^^11^ High-quality economic evaluations using prospective randomized clinical trials are needed to establish the relative costs, benefits, and cost-effectiveness of collagenase versus LF for DC.^12^

The Dupuytren’s Interventions: Surgery versus Collagenase (DISC) trial was a multicenter, pragmatic, 2-arm randomized clinical trial that was designed to compare collagenase and LF for treating moderate DC in patients aged 18 years or more in the United Kingdom.^13^ A total of 672 patients were recruited and randomized to receive either collagenase or LF. They were followed up at 2 and 6 weeks, 3 and 6 months, and 1- and 2-years after treatment. Details on the trial design are available in the published protocol.^13^ Patient and Public Involvement was included during the development, conduct and dissemination of the DISC Trial in which this evaluation was embedded. The findings on clinical effectiveness have been completed, peer reviewed, and are detailed in another publication.^14^

This article presents the results of a comprehensive economic evaluation conducted alongside the DISC trial to compare the short-term cost-effectiveness of collagenase with LF.^13^ A decision analytical model was also developed to evaluate the lifetime cost-effectiveness of the 2 treatments. This study aims to determine the value for money for both collagenase and LF over various time horizons, seeking to provide a robust foundation to inform the decision-making process.

Collagenase was withdrawn from the European market on February 29, 2020 and is currently only available in the United States.^15^ According to statements from both the European Medicines Agency and the International Dupuytren Society, the market withdrawal was due to commercial reasons rather than issues related to safety or health concerns.^15^^,^^16^ Therefore, this analysis offered additional evidence to inform potential decisions regarding collagenase if it were to become available again in the United Kingdom in the future.

## Methods

This economic evaluation was conducted from the perspective of the NHS and Personal Social Services, with the results presented in UK pounds (£) at 2019 to 2020 prices, reflecting the midpoint of the DISC trial and the timing of collagenase withdrawal from the UK market.^17^ The NHS Cost Inflation Index was used to adjust the costs from other years.^18^ The analysis was conducted in Stata 17.0 (within-trial analysis) and Microsoft Excel 2016 (long-term model).

### Within-Trial Cost-Effectiveness Analysis

To assess the short-term cost-effectiveness of collagenase compared with LF, a cost-effectiveness analysis (CEA) was undertaken using costs and health outcome data collected as part of the DISC trial.

#### Costs

A microcosting approach was used to calculate the costs associated with collagenase and LF.^19^ This included treatment delivery resources (eg, staff time, use of operating theatres, anesthesia and other medications) and patient healthcare resource use (eg, primary care, secondary care, and medications) collected by bespoke case report forms. The estimated costs were calculated by multiplying the quantity of each identified resource with their corresponding unit costs (see Appendix Table 1 in Supplemental Materials).^18^^,^^20^, ^21^, ^22^, ^23^, ^24^, ^25^

#### Health effects

Quality-adjusted life-years (QALYs) was the health outcome measure used in the CEA.^17^ Data collected via the EQ-5D-5L questionnaire were transformed into a single index value (ie, health utility) by mapping it to the EQ-5D-3L UK population valuation set.^26^ The utility scores derived from the baseline and subsequent follow-ups were used to calculate cumulative QALYs according to the area-under-the-curve method.^27^

#### CEA (base-case analysis)

An incremental CEA was performed by dividing the difference in the costs for collagenase and LF by the difference in QALYs to generate incremental cost-effectiveness ratios (ICERs).^28^ The ICERs were evaluated against the NICE-recommended willingness-to-pay (WTP) thresholds of £20 000 to £30 000 per QALY. We estimated the differences in expected costs and QALYs using seemingly unrelated regression equations, a widely used method to simultaneously estimate incremental costs and QALYs, accounting for potential correlation between these 2 outcomes.^29^ In clinical trials, both costs and QALYs typically exhibit nonnormal distributions, with costs often showing right-skewness and QALYs tending to be left-skewed.^30^ To account for these distributional features, a nonparametric bootstrap resampling method was applied to estimate 95% confidence intervals (CIs) for incremental costs, incremental QALYs, and ICERs.^31^^,^^32^ Cost-effectiveness acceptability curves (CEACs) were used to illustrate the probability of collagenase being cost-effective at various WTP thresholds. The results also presented net health benefits (NHBs), which is a useful metric when an intervention is less costly but produces fewer health benefits.^17^ NHB is calculated as: NHB = (Incremental QALYs × WTP threshold) - Incremental costs. A positive NHB indicates that the intervention is cost-effective at the given WTP threshold because the health gains outweigh the incremental costs. Conversely, a negative NHB indicates that the intervention is not cost-effective.^17^

Multiple imputation was used to account for missing data assuming that the unobserved data were missing at random.^33^ Missing data were imputed using multiple imputation by chained equations.^34^ The imputation model was developed based on expert knowledge, prior research, and analysis of the trial data set. It included variables used in the CEA and additional auxiliary variables associated with missingness.^34^ Logistic regression analyses (for continuous and binary variables) and chi-square tests (for categorical variables) were conducted to identify candidate variables significantly associated with missingness in either costs or QALYs. Additionally, key variables expected to predict costs or QALYs, such as treatment allocation, baseline costs, and utility scores, were included irrespective of their statistical significance.

The final imputation model includes primary outcomes (costs and utility scores at baseline and at each follow-up), demographic factors (age and gender), condition severity indicators (number of cords affected), participant trial status (ongoing participation, withdrawal, or deceased), and hand-related outcome measures (Patient Evaluation Measure). The imputation was repeated 25 times, and the imputed data sets were used in the base-case analysis. Deceased participants were assigned 0 utility scores and costs at all scheduled follow-up points occurring after their date of death.^35^

#### Sensitivity and secondary analyses

A series of sensitivity analyses were conducted to examine the robustness of the base-case analysis. An analysis using only complete cases was undertaken to obtain results under a slightly different missing at random assumptions for the unobserved data. Healthcare Resource Groups (HRGs) reference costs were used to calculate the intervention cost to enable comparison with other studies using the same approach.^4^^,^^36^ The HRG codes used for LF, wound clinic, collagenase injection, and manipulation comprised HN43B (unit cost £2936), HN46Z (£196), HN45A (£1143), and HN46Z (£196), respectively.^20^ The impact of different treatment locations on cost-effectiveness was assessed assuming that collagenase injections were performed in an operating theatre, which is the case for LF, instead of in an outpatient setting.^37^ Staff delivering LF were assumed to be trainee surgeons instead of consultant surgeons, as is current practice.^21^^,^^38^ A secondary analysis was conducted from a wider societal perspective to examine whether the cost-effectiveness of collagenase compared with LF may be affected by time away from work and routine activities.^39^ A threshold analysis was also performed to explore the maximum acceptable price that would result in collagenase being considered cost-effective.

### Long-Term Markov Decision Analytic Model

#### Model structure

**Figure 1:**
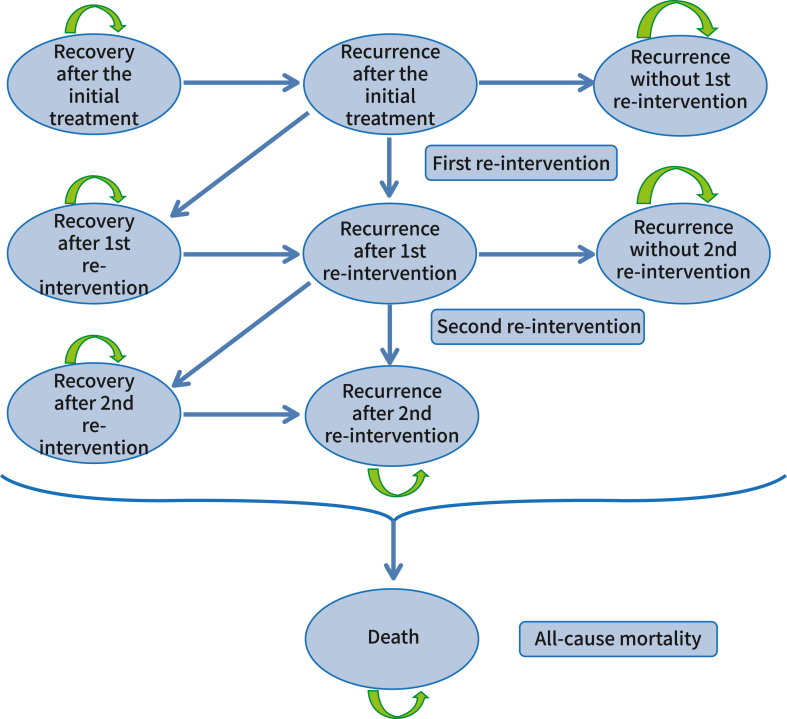
Structure of the Markov model.

To explore the long-term cost-effectiveness of collagenase versus LF, a Markov model was developed based on previously published models that compared the treatments for DC.^40^^,^^41^ The Markov model was used to simulate the health state progressions of the cohort of patients from the DISC trial. The model consisted of 9 mutually exclusive health states, shown in Figure 1. The starting point of the model was set at 1-year after treatment. Based on the 1-year trial data, patients entered the model in either the “recovery after initial correction” or “recurrence after initial correction” state. After this, they could either transition to other health states or stay in their current state, as indicated by the arrows. Based on the corresponding reintervention rate, a proportion of patients who had experienced recurrence after initial treatment were eligible for up to 2 reinterventions. The model period was set to a 1-year cycle length, and the process continued until the patients reached the age of 85.

#### Model inputs

Model inputs and sources are detailed in Appendix Table 2 in Supplemental Materials. The transition probabilities for moving between the health states were derived from both the DISC trial and the existing literature. Transition probabilities for the first year after treatment were derived from the trial data. For subsequent years (year 2 and onward), data were synthesized from studies with the longest available follow-up periods to calculate annual rates that more accurately reflect long-term clinical outcomes. Point estimates of recurrence rates 1-year posttreatment were 13.8% for LF and 17.2% for collagenase based on trial data. For the second year and subsequent years, annual recurrence rates derived from earlier long-term studies were 11.8% for collagenase and 5.4% for LF.^9^^,^^42^, ^43^, ^44^, ^45^, ^46^ Patients experiencing recurrence may opt for reintervention. Because of the impact of the COVID-19 pandemic, only 12 patients reported having undergone reintervention for DC during the trial, which is lower than the rates cited in other studies.^47^ To simplify the model, a one-off reintervention rate of 40% for both the first and second reinterventions was assumed, following published models.^40^^,^^48^ Patients in every health state were at risk of dying at the end of each cycle, with the all-cause mortality rates being obtained from the Office for National Statistics death registry by age and gender.^49^

Each health state cycle is associated with specific healthcare costs and health outcomes (utilities). Average costs and QALYs from the first year were used as inputs for the initial year after treatment or reintervention. Given the differences in clinical pathways between collagenase injections and LF surgery, treatment-specific values were applied throughout the model’s time horizon. Evidence from existing literature report differences in clinical outcomes between these 2 treatments when assessed beyond 5 years after treatment.^50^^,^^51^ We assumed that the second year posttreatment data represented a stable long-term state and used the annual costs and QALYs from this period as model input for second year after treatment and onward in the model.

It was also assumed that the costs associated with reintervention would be equivalent to those of the initial intervention.

#### CEA and probabilistic sensitivity analysis

An incremental CEA was conducted to compare the lifetime cost-effectiveness of the 2 interventions. An annual discount rate of 3.5% was applied to all costs and QALYs.^17^ To quantify the uncertainty of the model’s parameters, a probabilistic sensitivity analysis was performed using Monte Carlo simulation.^52^ This involved generating 10 000 iterations of lifetime costs and QALYs based on the values of the parameters that were randomly selected from the preselected distributions according to the type of data (see Appendix Table 2 in Supplemental Materials).

## Results

### Costs and Health Benefits

There were 336 patients in each trial group, with a mean intervention cost of £2166 (95% CI £2142 to £2191) for LF and £984 (95% CI £979 to £988) for collagenase (Table 1). Costs associated with healthcare resource use were slightly higher in the LF group at 1 year (mean difference: £91, 95% CI £50 to £133), but no significant difference was detected over a 2-year time horizon. Details on the breakdown of costs can be found in Appendix Tables 3 and 4 in Supplemental Materials. The distributions of costs and QALYs for both trial groups at the 1-year and 2-year follow-ups are illustrated in Appendix Figure 1 in Supplemental Materials.

**Table 1:** Cost-effectiveness analysis results (multiple imputation).

Outcome	LF group (*N* = 336)	Collagenase group (*N* = 336)	Mean difference∗^,^†^,^‡^,^§
1-year results			
Intervention cost, mean (95% CI)	£2166 (£2142-£2191)	£984 (£979-£988)	−£1183 (−£1208 to −£1159)
Healthcare resource use cost, mean (95% CI)	£521 (£502-£540)	£613 (£576-£650)	£91 (£50-£133)
Total cost, mean (95% CI)	£2688 (£2656-£2719)	£1596 (£1559-£1634)	−£1090 (−£1139 to −£1042)
Effect (QALY), mean (95% CI)	0.838 (0.835-0.840)	0.833 (0.830-0.836)	−0.003 (−0.006 to 0.0004)
ICER	Collagenase is less costly and less effective.		
NHB	0.052 (at £20 000/QALY)		0.033 (at £30 000/QALY)
Probability of collagenase being cost-effective	100.0% (at £20 000/QALY)		99.9% (at £30 000/QALY)
2-year results			
Intervention cost, mean (95% CI)	£2166 (£2142-£2191)	£984 (£979-£988)	−£1183 (−£1208 to −£1159)
Healthcare resource use cost, mean (95% CI)	£914 (£872-£956)	£884 (£842-£926)	−£28 (−£87 to £30)
Total cost, mean (95% CI)	£3081 (£3032-£3129)	£1868 (£1825-£1910)	−£1212 (−£1276 to −£1147)
Effect (QALY), mean (95% CI)	1.687 (1.682-1.693)	1.636 (1.629-1.643)	−0.048 (−0.055 to −0.040)
ICER	Collagenase is less costly and less effective.		
NHB	0.013 (at £20 000/QALY)		−0.007 (at £30 000/QALY)
Probability of collagenase being cost-effective	71.9% (at £20 000/QALY)		37.0% (at £30 000/QALY)

ICER indicates incremental cost-effectiveness ratio; NHB, net health benefit; QALY, quality-adjusted life-year.

∗ Deaths included as 0.

† Adjusted for baseline cost and utilities.

‡ CIs are based on 5000 bootstraps (ie, 200 bootstraps for each of the 25 imputed data sets).

§ Costs are rounded to the nearest pound and QALYs are rounded to 3 decimal places.

Appendix Table 5 in Supplemental Materials shows the mean EQ-5D-5L utility scores at baseline and each follow-up. Both groups experienced a notable worsening in quality of life immediately after the trial treatment, but the LF group was particularly affected. At 2 weeks after treatment, the LF group’s utility scores decreased from 0.794 to 0.715, whereas the collagenase group’s utility scores dropped from 0.791 to 0.776. However, 6 weeks after treatment, as the participants began to recover, the utility scores also started to recover. At both the 2 and 6 weeks, the collagenase group had significantly higher utility scores than the LF group (mean difference at 2 weeks: 0.061, 95% CI 0.037-0.085; at 6 weeks: 0.03, 95% CI 0.005-0.055). At 3 months, both groups reported an improved quality of life compared with pretreatment. However, although the LF group’s utility scores stabilized after 3 months, those of the collagenase group steadily worsened over time, with the collagenase group’s mean utility scores being significantly lower by the end of the 2 years (mean difference: −0.044, 95% CI −0.077 to −0.010).

### Within-Trial Cost-Effectiveness

#### Base-case CEA

**Figure 2:**
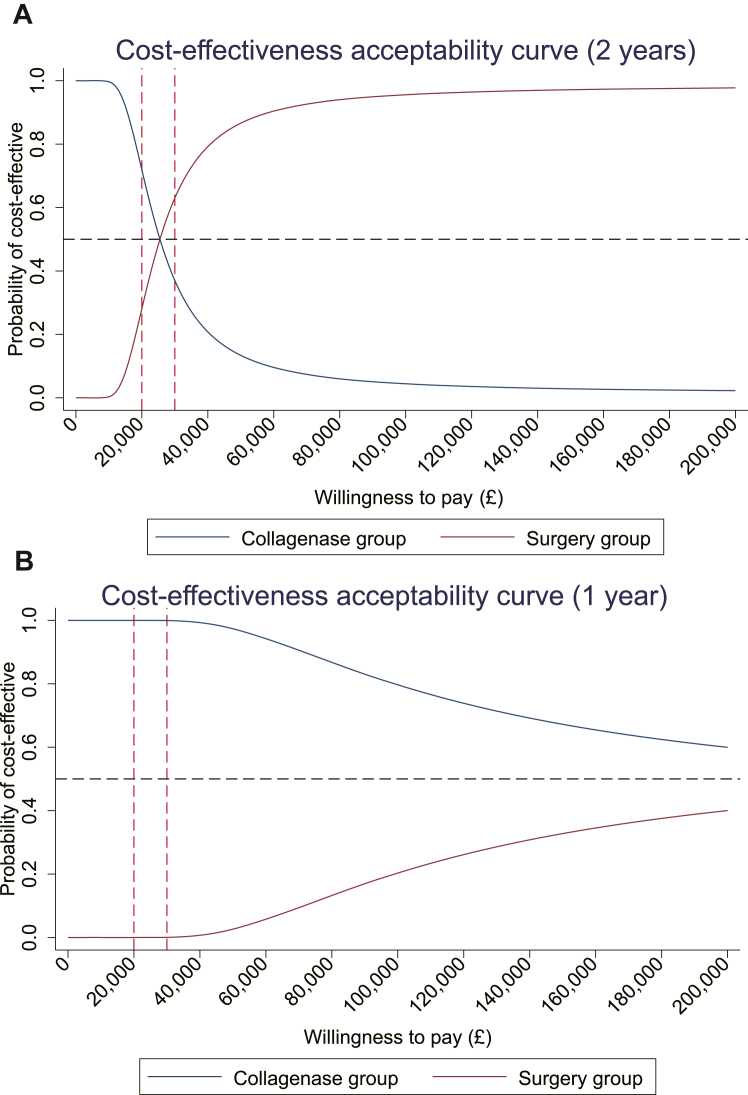
(A) Cost-effectiveness acceptability curve (1 year). (B) Cost-effectiveness acceptability curve (2 years).

Table 1 provides the base-case CEA results using the multiple imputation data set. After adjusting for baseline costs and utilities, the collagenase group showed an insignificantly lower QALY gain of −0.003 (95% CI −0.006 to 0.0004) and a significantly lower total cost compared with the LF group within the first year: −£1090 (95% CI −£1139 to −£1042). This cost saving was mainly a result of the considerably lower intervention cost. At the WTP thresholds of £20 000 and £30 000 per QALY, the NHBs of collagenase were estimated to be 0.052 and 0.033. The CEAC shown in Figure 2A indicates that, at 1 year, the probability of collagenase being cost-effective exceeds 99% for WTP thresholds ranging from £20 000 to £30 000 per QALY.

Repeating the above analysis for the period of 2 years after treatment found that collagenase still offered significant cost savings compared with LF (mean difference: −£1212, 95% CI −£1276 to −£1147). However, the QALY gains at 2 years were significantly lower than those of the LF group −0.048 (−0.055 to −0.040). At a WTP threshold of £20 000 per QALY, the 2-year CEAC (Figure 2B) shows that the probability of collagenase being cost-effective compared with LF was 72%. However, as the WTP threshold increased, the probability of collagenase being cost-effective decreased. When the WTP exceeded £25 488, LF became the optimal treatment choice.

#### Sensitivity and secondary analyses

Table 2 lists the results of the sensitivity and secondary analyses conducted for both 1 year and 2 years after treatment, which demonstrated that collagenase was always the significant cost-saving option. At the 1-year follow-up, collagenase had slightly lower QALY gains that were statistically insignificant compared with LF, and it consistently yielded positive NHBs. The probability of collagenase being cost-effective was more than 95%. These findings were robust across all additional analyses.

**Table 2:** Results of secondary and sensitivity analyses.

	Collagenase vs LF			Probability of collagenase being cost-effective at specified WTP threszhold per QALY		NHB associated with collagenase at specified WTP threshold per QALY	
Analysis	Incremental cost mean (95% CI)∗^,^†^,^‡	Incremental QALYs mean (95% CI)∗^,^†^,^‡	ICER	£20 000	£30 000	£20 000	£30 000
Base-case analysis (multiple imputation)			Less costly and less effective				
1 year (*N* = 672)	−£1090 (−£1139 to −£1042)	−0.003 (−0.006 to 0.000)		100.0%	99.9%	0.052	0.033
2 years (*N* = 672)	−£1212 (−£1276 to −£1147)	−0.048 (−0.055 to −0.040)		71.9%	37.0%	0.013	−0.007
Secondary analysis (wider societal perspective)							
1 year (*N* = 672)	−£1363 (−£1431 to −£1295)	−0.001 (−0.004 to 0.003)		100.0%	100.0%	0.067	0.045
2 years (*N* = 672)	−£1458 (−£1543 to −£1374)	−0.041 (−0.048 to −0.033)		91.9%	64.3%	0.032	0.008
Sensitivity analyses							
Complete cases							
1 year (*N* = 361)	−£1374 (−£1640 to −£1109)	−0.009 (−0.030 to 0.011)		100.0%	99.9%	0.059	0.036
2 years (*N* = 285)	−£1505 (−£1988 to −£1022)	−0.060 (−0.113 to −0.007)		69.7%	37.0%	0.015	−0.010
HRG cost							
1 year (*N* = 672)	−£1320 (−£1367 to −£1273)	−0.0001 (−0.003 to −0.003)		100.0%	100.0%	0.066	0.044
2 years (*N* = 672)	−£1434 (−£1496 to −£1371)	−0.042 (−0.050 to −0.035)		91.8%	60.8%	0.029	0.006
Collagenase injections in theatre							
1 year (*N* = 672)	−£528 (−£576 to −£480)	−0.003 (−0.006 to 0.000)		98.9%	94.6%	0.023	0.014
2 years (*N* = 672)	−£648 (−£712 to −£584)	−0.048 (−0.055 to −0.041)		29.6%	14.0%	−0.015	−0.026
Limited fasciectomy delivered by trainees							
1 year (*N* = 672)	−£985 (−£1033 to −£938)	−0.002 (−0.006 to 0.001)		100.0%	99.9%	0.047	0.030
2 years (*N* = 672)	−£1108 (−£1172 to −£1044)	−0.046 (−0.053 to −0.038)		66.3%	34.5%	0.010	−0.009

HRG indicates Healthcare Resource Group; ICER, incremental cost-effectiveness ratio; LF, limited fasciectomy; NHB, net health benefit; QALY, quality-adjusted life-year; WTP, willingness-to-pay.

∗ Adjusted for baseline EQ-5D utility and baseline cost.

† CIs are based on 5000 bootstraps.

‡ Costs are rounded to the nearest pound and QALYs are rounded to 3 decimal places.

In contrast, the results at 2 years were more sensitive to varying assumptions. During the DISC trial, there were 8 recorded deaths in the collagenase group compared with only 1 in the LF group. Testing an alternative costing method using the HRG reference cost increased the potential cost savings associated with collagenase and raised the probability of it being cost-effective to 92% and 61% at WTP thresholds of £20 000 and £30 000 at 2 years, respectively. Because using operating theatres is a major cost driver for LF treatment, 1 sensitivity analysis showed that if all collagenase injections were performed in an operating theatre, LF would become the cost-effective option at 2 years. Downgrading surgical staff from consultant surgeons to trainees did not affect the results, with findings being consistent with the results of the base-case analyses.

The secondary analysis, conducted from a broader societal perspective, showed significantly higher productivity loss in the LF group due to absence from work after treatment compared with the collagenase group (£487 vs £138; mean difference: −£350, 95% CI −£537 to −£162). This increased overall cost for LF and resulted in collagenase being considered more cost-effective, with probabilities of 92% (at £20 000) and 64% (at £30 000) at 2 years. Appendix Figure 2 in Supplemental Materials depicts the threshold analysis on collagenase’s cost-effectiveness relative to its price. At 2 years, collagenase is cost-effective if priced under £910 per vial at a WTP threshold of £20 000 per QALY and under £300 per vial at £30 000 per QALY.

### Markov Model Results

Table 3 presents the Markov model results based on the data from the 345 participants who had recorded recurrent status at the 1-year follow-up. The Markov model included the option for reintervention for patients experiencing recurrence, which may have been previously missed because of the pandemic. At 2 years, the probability of collagenase being the optimal strategy increased from 37% (within-trial results) to 69% (model-based results) at a WTP threshold of £30 000 per QALY. However, over a longer term, the likelihood of collagenase being cost-effective diminished, with LF becoming the most cost-effective option from the fourth year onward.

**Table 3:** Markov model results.

	LF		Collagenase		Incremental cost∗	Incremental QALYs∗	ICER	Probability of collagenase being cost-effective at specified WTP threshold per QALY		NHB associated with collagenase at specified WTP threshold per QALY	
Time post initial intervention	Mean cost	Mean QALY	Mean cost	Mean QALY				£20 000	£30 000	£20 000	£30 000
1 year (*N* = 345)	£3025	0.846	£1535	0.835	−£1490	−0.011	Collagenase less costly and less effective	100.0%	99.7%	0.064	0.039
2 years (*N* = 345)	£3479	1.688	£1838	1.633	−£1641	−0.054		94.3%	69.3%	0.028	0.0005
3 years (*N* = 345)	£3897	2.481	£2112	2.386	−£1784	−0.096		64.1%	36.7%	−0.007	−0.036
4 years (*N* = 345)	£4275	3.230	£2357	3.095	−£1918	−0.135		45.1%	25.2%	−0.039	−0.071
Lifetime (*N* = 345)	£7008	9.610	£4040	9.126	−£2968	−0.484		21.9%	16.2%	−0.335	−0.385

ICER indicates incremental cost-effectiveness ratio; LF, limited fasciectomy; NHB, net health benefit; QALY, quality-adjusted life-year; WTP, willingness-to-pay.

∗ Costs are rounded to the nearest pound and QALYs are rounded to 3 decimal places.

Over a patient’s lifetime, collagenase had an estimated total cost saving of £2968 per patient when compared with LF, but this was associated with a mean QALY loss of −0.484. At WTP thresholds of £20 000 and £30 000 per QALY, the probability that collagenase would be considered cost-effective in comparison with LF at the lifetime horizon dropped below 22% and 16%, respectively.

## Discussion

A recent longitudinal study from the United Kingdom reported a growing incidence of DC over recent decades.^53^ Although a variety of treatment options exist, surgical procedures, such as LF, continue to be commonly used because of their favorable long-term outcomes and comparatively lower recurrence rates in clinical practice, despite their invasive nature.^54^ In contrast, nonsurgical approaches, such as collagenase injections, provide advantages in terms of being less invasive and offering quicker recovery times, but they yield less durable outcomes.^55^ However, collagenase is a newer therapeutic approach for DC, with limited cost-effectiveness evidence available when compared with other established treatments. We conducted a full CEA alongside the DISC trial and also developed a decision analytical model to assess both short- and long-term cost-effectiveness of collagenase versus LF, addressing gaps in the current literature.

Overall, collagenase is a less costly and less effective intervention compared with LF. One-year after treatment, collagenase demonstrated a high probability of being cost-effective compared with LF. This indicates that with constrained resources, collagenase could improve overall population health by releasing resources used for operating on DC.^56^^,^^57^ One-year cost-effectiveness results for collagenase were robust across all secondary and sensitivity analyses, with its probability of being cost-effective consistently exceeding 95%.

At 2 years after treatment, although collagenase remained the more cost-effective option at lower WTP thresholds, LF became the optimal treatment when the thresholds exceeded £25 488 per QALY. At 2 years, there was a considerable level of uncertainty surrounding the results, with the base-case conclusions heavily influenced by alterations in the costing methods in the secondary and sensitivity analyses. Sensitivity analyses revealed that assumptions reducing collagenase costs or increasing LF costs improved collagenase’s cost-effectiveness, whereas the opposite was true if LF costs were reduced, or collagenase costs were increased. Overall, these 2-year sensitivity analyses did not yield a definitive conclusion on cost-effectiveness.

The results from the Markov model suggest that the likelihood of collagenase being cost-effective decreases with time. When considering the lifetime horizon, the probability of collagenase being cost-effective compared with LF is only around 20% at WTP thresholds above £20 000. This aspect is partially explained by the heterogeneity observed in published studies, with differing results seen potentially arising from the varying time horizons investigated.^58^ The model-based CEA comparing collagenase with LF for the treatment of DC similarly indicated a time-dependent trend, favoring collagenase at 1 year, consistent with the within-trial findings. To account for potential disruptions in care during the DISC trial related to the COVID-19 pandemic, which may have delayed or postponed reinterventions, our Markov model incorporated a 40% reintervention rate for participants experiencing recurrence. Under these assumptions, the model-based results at 2 years showed an increased probability of collagenase being cost-effective compared with the direct within-trial findings at the same time point.

Most existing cost-effectiveness studies are retrospective and usually cover short time horizons, and many of them report collagenase as a cost-effective treatment in comparison with others.^21^^,^^59^, ^60^, ^61^, ^62^ In contrast, studies adopting longer time horizons tend to favor LF. A systematic review by Fitzpatrick et al^10^ identified that only 2 of the 17 included studies concluded LF was cost-effective, both using time horizons extending beyond 10 years.^40^^,^^63^ Specifically, Sau et al^63^ applied a 10-year horizon, whereas Brazzelli et al^40^ used a lifetime horizon consistent with our current analysis. The results from these studies, including this study, consistently indicate that LF is more cost-effective compared with collagenase over long term. These conclusions align with the NICE Technology Appraisal (TA459), which similarly reported that collagenase was dominated by LF.^64^ However, both NICE TA459 and Brazzelli et al^40^ relied on naive indirect comparisons because of the absence of head-to-head randomized controlled trials at the time, a limitation specifically acknowledged by NICE as a significant source of uncertainty. Therefore, this study enhances the existing evidence base by providing direct comparative data from a robust randomized trial, thereby increasing its reliability and applicability for clinical decision making and policy recommendations.

DC impairs patients’ quality of life significantly, and patients from both groups reported worsened quality of life soon after receiving the treatment, especially in the LF group.^7^^,^^11^^,^^65^ After the transitory posttreatment decline, EQ-5D utility scores were consistently higher in the LF group from 3 months onward. The significant initial drop, followed by subsequent higher scores in the LF group, resulted in a small and insignificant increase in QALYs (0.003) compared with collagenase group. Given the substantial cost savings in the collagenase group, collagenase was determined to be highly cost-effective at 1 year. However, when viewed over a longer time frame, the LF group demonstrated a significant increase in QALYs accumulated over time, resulting in significantly higher mean QALYs (0.048) by the end of 2 years compared with the collagenase group. The significant cost savings in the collagenase group and the higher QALYs in the LF group lead to increased uncertainty around the cost-effectiveness, making the decision less conclusive at 2 years compared with 1 year.

The long-term model developed in this study included potential recurrences beyond the trial’s observation period. It also incorporates the possibility of reinterventions after a recurrence, an option that patients might have missed because of COVID-19 restrictions during the trial period. The results suggest that timely reinterventions enhance the cost-effectiveness of collagenase at 2 years, compared with the within-trial findings. However, this effect is short-lived, and over time LF emerges as the more cost-effective option.

Adopting a microcosting method, this study provided detailed costs associated with the provision of the collagenase and LF treatment to DC patients. The results of this study identified key cost drivers for both interventions, providing valuable guidance for subsequent intervention development. It also highlighted the need to scrutinize costing methods and individual cost components when comparing the cost-effectiveness of identical interventions across different studies. The heterogeneity in reported cost-effectiveness of collagenase compared with LF is notable across previous studies, largely attributed to inconsistency in study design and the types of costs considered.^10^^,^^11^

The impact of alternative costing methods and major cost drivers on overall cost-effectiveness were tested in sensitivity analyses in this study. Using the HRG reference cost, which yielded greater cost savings for collagenase compared with microcosting findings, the results favored collagenase. In contrast, when the setting for collagenase injections shifted from outpatient to an operating theatre, which reduced cost savings from collagenase, LF became the cost-effective treatment at 2 years, demonstrating the significance of location on the cost-effectiveness of these interventions.^21^^,^^37^ The cost of collagenase constituted the largest cost component of the collagenase injection intervention and this price was found to affect cost-effectiveness in the threshold analysis.^66^^,^^67^ The extensive sensitivity analyses provided potential strategies for improving the cost-effectiveness of either intervention in the future.

Both collagenase and LF have their own merits and limitations in relation to healthcare resource use. LF-treated patients had significantly more posttreatment outpatient visits for physical therapy and also reported greater short-term productivity loss due to absences from work and other activities, consistent with findings from prior studies.^21^^,^^39^^,^^59^ In contrast, patients receiving collagenase exhibited higher in-hospital costs stemming from an elevated number of supplementary hand treatments, reflecting a higher recurrence rate compared with the LF group.^9^^,^^42^^,^^44^ Furthermore, although 86% of collagenase patients had a single joint treated at a time, 40% of LF patients received treatment on multiple digits simultaneously. Therefore, if the analysis was based on digits treated rather than patients, the cost-effectiveness of LF would likely be enhanced, given that the marginal cost for treating additional digits would be lower than the initial digit.^68^

Limitations of the study included omissions in data collection during follow-ups and delayed reinterventions for participants because of COVID-19 restrictions. However, the Markov model incorporated potential reinterventions based on data from the published literature. It is important to note that the model’s outcomes carry uncertainty due to assumptions about model structure and model inputs that simplify the complexity of clinical reality. The model’s cost and utility inputs relied heavily on data collected during the 2-year trial period, given that robust, long-term comparative data were unavailable.

An extended trial follow-up would help refinement of the model and validation of results. It is crucial to use a sufficiently long follow-up period when comparing surgical and nonsurgical interventions in a trial, in which the recovery and posttreatment maintenance of correction display distinctive patterns over time. This will help accurately capture the true overall costs and benefits of the interventions, offering decision makers robust evidence for optimal long-term healthcare resource allocation. Future updates and refinements of the Markov model are also recommended once long-term trial outcomes and real-world evidence become available.

## Conclusions

Collagenase is less costly and less effective than LF. It is highly cost-effective at 1 year because of lower intervention costs and quicker recovery. However, the probability of collagenase being cost-effective declined over time because of higher recurrence rates and additional treatments. The Markov model indicates that LF is more cost-effective over a lifetime horizon. The results suggest the need for longer trial follow-ups when comparing surgical with nonsurgical interventions to fully capture their long-term costs and benefits.

## Article and Author Information

**Authorship Confirmation:** All authors certify that they meet the ICMJE criteria for authorship.

**Collaborators: DISC Trial Team:** Maria Armaou, PhD (TMG member, University Hospitals of Leicester NHS Trust, Leicester, England, UK); Christopher Bainbridge, MB ChB (TMG member, Co-applicant, University Hospitals of Derby and Burton NHS Trust, Derby, England, UK); John Cooke, BA (TMG member, PPI representative, Leicester, England, UK); Michael Craigen, MB BS (TMG member, Co-applicant, The Royal Orthopaedic Hospital, Birmingham, England, UK); Lydia Flett, MPH (TMG member, York Trials Unit, Department of Health Sciences, University of York, York, England, UK); Samantha Brady, PhD (TMG member, York Trials Unit, Department of Health Sciences, University of York, York, England, UK); Catherine Hewitt, PhD (TMG member, Co-applicant, York Trials Unit, Department of Health Sciences, University of York, York, England, UK); Sophie James, MSc (TMG member, York Trials Unit, Department of Health Sciences, University of York, York, England, UK); Judy Jones (TMG member, University Hospitals of Leicester NHS Trust, Leicester, England, UK); Ada Keding, MSc (TMG member, Co-applicant, York Trials Unit, Department of Health Sciences, University of York, York, England, UK); Catherine Knowlson, PhD (TMG member, York Trials Unit, Department of Health Sciences, University of York, York, England, UK); Paul Leighton, PhD (TMG member, Co-Applicant, University of Nottingham, Nottingham, England, UK); David A. Torgerson, PhD (TMG member, Co-Applicant, York Trials Unit, Department of Health Sciences, University of York, York, England, UK); David Warwick, MD (TMG member, Co-Applicant, University Hospital Southampton NHS Foundation Trust, Southampton, England, UK); Michelle Watson, MSc (TMG member, York Trials Unit, Department of Health Sciences, University of York, York, England, UK).

**Funding/Support:** This research was funded by the 10.13039/501100000272National Institute for Health and Care Research (NIHR) Health Technology Assessment programme (Reference: 15/102/04).

**Role of the Funder/Sponsor:** The funder had no role in the design and conduct of the study; collection, management, analysis, and interpretation of the data; preparation, review, or approval of the manuscript; or decision to submit the manuscript for publication.

## Author Disclosures

Author disclosure forms can be accessed below in the Supplemental Material section. The views and opinions expressed therein are those of the authors and do not necessarily reflect those of the Health Technology Assessment programme, NIHR, NHS or the Department of Health.

## Supplemental Material

Author Disclosures
