# Supplementary material for: The Cost-Effectiveness of Collagenase Injection Versus Limited Fasciectomy for Moderate Dupuytren’s Contracture: An Economic Evaluation of the Dupuytren’s Interventions Surgery Versus Collagenase Trial and a Decision Analytical Model
Source: Value Health. 2026 Jan;29(1):54–63. doi: 10.1016/j.jval.2025.07.030 (PMC12757186; doi:10.1016/j.jval.2025.07.030)
Supplement: Supplemental Material [file mmc2.docx]

# Supplementary information

Table 1: Unit costs (2019/20) used in the analyses

| Resource item | Unit cost (2019/20) | Source |
| --- | --- | --- |
| Intervention-related cost items | | |
| Staff | | |
| Consultant surgeon | £114/hour | [1] |
| Trainee surgeon | £50/hour | [1] |
| Nurse | £46/hour | [1] |
| Healthcare assistant | £27/hour | [1] |
| Operating department practitioner | £46/hour | [1] |
| Anaesthesia |  |  |
| Anaesthesia—general anaesthetic | £193/session | [2] |
| Anaesthesia—local anaesthetic | £124/session | [2] |
| Anaesthesia—regional block | £124/session | [2] |
| Anaesthesia—Entonox | £124/session | [2] |
| Anaesthesia—sedation | £193/session | [2] |
| Anaesthesia—other | £124/session | [2] |
| Theatre cost | £15/minute | [3] |
| Inpatient cost | £996/night | [2, 4] |
| Antibiotics |  |  |
| Cephalosporins | £3.4/item | [5] |
| Teicoplanin | £61.5/item | [5] |
| Macrolides (Erythromycin) | £5.8/item | [5] |
| Co-amoxiclav (Augmentin) | £3.0/item | [5] |
| Vancomycin hydrochloride | £239.7/item | [5] |
| Other antibiotics | £23.1/item | [5] |
| Other medication | £8.55/item | [5] |
| Collagenase |  |  |
| Collagenase before 31/08/2018* | £650/vial | [6] |
| Collagenase after 01/09/2018 | £572/vial | [7] |
| Healthcare resource use | | |
| Primary care |  |  |
| Visit to GP | £39/consultation | [1] |
| Visit to GP nurse | £11/consultation | [1] |
| Physiotherapist | £57/session | [1] |
| Occupational therapist | £84/session | [1] |
| Secondary care | | |
| Outpatient visit |  |  |
| Pain clinic | £182/session | [2] |
| Trauma & orthopaedics | £122/session | [2] |
| Occupational therapy | £72/session | [2] |
| Trauma & orthopaedics | £122/session | [2] |
| Plastic surgery | £117/session | [2] |
| Rheumatology | £146/session | [2] |
| Physiotherapy | £62/session | [2] |
| Splinting of finger | £84/session | DISC |
| Wound care | £15/session | DISC |
| Collagenase injection | £1143/session | [2] |
| LF surgery | £2936/session | [2] |
| Dermo-fasciectomy | £2936/session | [2] |
| PNF | £1,143/session | [2] |
|  |  |  |
| Accident and emergency | £182/attendance | [2] |
| Hospital inpatient | £996/night | [2, 4] |
| Day case | £812/episode | [2] |
| Emergency ambulance | £213/use | [2] |

* The unit cost for collagenase was reduced from £650 to £572 per vial effective from 1st September 2018; the revised price was adopted in the base-case analysis.

Table 2: Model inputs and sources

| Variable | Limited fasciectomy group | Collagenase group | Distribution (SE) | Source |
| --- | --- | --- | --- | --- |
| Baseline demographics |  |  |  |  |
| Mean age | 66 | 66 |  | DISC |
| Male (%) | 76.8% | 81.5% |  | DISC |
| Transition probabilities |  |  |  |  |
| Recurrence rate (1st year post-treatment) | 13.8% | 17.2% | β (2.6% & 2.8%) | DISC |
| Recurrence rate (2nd year and beyond post-treatment) | 5.4% | 11.8% | β (1.1% & 2.0%) | [8-12] |
| 1st Re-intervention rate | 40% | 40% | β (15% & 15%) | [12, 13] |
| 2nd Re-intervention rate | 40% | 40% | β (15% & 15%) |  |
|  |  |  |  |  |
| Mortality |  |  |  |  |
| 65-69 (Males) | 1.60% | 1.60% |  | [14] |
| 70-74 (Males) | 2.48% | 2.48% |  |  |
| 75-79 (Males) | 4.32% | 4.32% |  |  |
| 80-84 (Males) | 7.70% | 7.70% |  |  |
| 65-69 (Females) | 1.00% | 1.00% |  |  |
| 70-74 (Females) | 1.62% | 1.62% |  |  |
| 75-79 (Females) | 2.91% | 2.91% |  |  |
| 80-84 (Females) | 5.46% | 5.46% |  |  |
|  |  |  |  |  |
| Mean costs and QALYs |  |  |  |  |
| Treatment cost | £2,510 | £1,008 | γ (£69 & £9) | DISC |
| 1st year post-treatment cost of healthcare source use (recovery) | £620 | £578 | γ (£84 & £116) | DISC |
| 1st year post-treatment cost of healthcare source use (recurrence) | £323 | £281 | γ (£94 & £57) | DISC |
| 2nd year post-treatment cost of healthcare source use (recovery) | £340 | £239 | γ (£116 & £139) | DISC |
| 2nd year post-treatment cost of healthcare source use (recurrence) | £267 | £166 | γ (£57 & £61) | DISC |
| 1st year post-treatment QALY (recovery) | 0.847 | 0.836 | β (0.012 & 0.011) | DISC |
| 1st year post-treatment QALY (recurrence) | 0.841 | 0.831 | β (0.027 & 0.019) | DISC |
| 2nd year post-treatment QALY (recovery) | 0.865 | 0.819 | β (0.015 & 0.015) | DISC |
| 2nd year post-treatment QALY (recurrence) | 0.852 | 0.806 | β (0.041 & 0.026) | DISC |

## Breakdown of intervention costs and healthcare resource use costs

In the trial, a total of 288 patients underwent LF treatment, and 325 patients received collagenase. A detailed breakdown of the intervention costs involved in each treatment is provided in (Table 3). On average, the LF treatment cost was estimated at £2,510 per patient (SD £818), while the collagenase treatment was less expensive at an average of £1,008 per patient (SD £94). The most substantial component of the LF cost (66%) was attributed to the use of the operating theatre. In contrast, the cost of the collagenase accounted for the majority of the collagenase cost (58%). The second most significant expense for both the LF and collagenase treatments was staff costs. In the cost-effectiveness analysis, participants were analysed according to their randomised allocation, following the intention-to-treat principle.

Table S4 provides a breakdown of the average costs associated with the healthcare resource use for each component at each follow-up. Overall, the collagenase group had higher healthcare costs than the LF group during the initial three months and at the second-year post-treatment follow-up. However, the collagenase group’s costs were lower at the six-month and one-year follow-ups. There were no statistically significant differences in the cost of the total healthcare resource use at any of the time points. However, during the first three months post-treatment, the LF patients incurred significantly higher costs in their primary care, which included the costs for physiotherapists, occupational therapists, and outpatient wound care and physiotherapy.

Table 3: Intervention cost by treatment

|  | Mean resource use (SD) | Unit | Mean cost (SD) | Range |
| --- | --- | --- | --- | --- |
| Surgery |  |  |  |  |
|  | Number of participants who received surgery (N=288) | | | |
| Theatre cost | 110 (39) | Minute | £1,657 (£580) | (£531 to £3,900) |
| Inpatient cost | 0.01(0.1) | Night | £14 (£143) | (£0 to £1,992) |
| Staff cost (Surgery) | 109 (46) | Minute | £560 (£237) | (£101 to £1,776) |
| Anaesthetic cost | 1.44 (0.6) | Session | £213 (£87) | (£124 to £441) |
| Antibiotics cost | 2.3 (7.6) | Item | £2 (£7) | (£0 to £62) |
| Additional meds | 0 (1) | Item | £0 (£1) | (£0 to £9) |
| Staff cost (Wound care) | 32 (31) | Minute | £64 (£61) | (£0 to £483) |
| Total surgery cost |  |  | **£2,510 (£818)** | **(£866 to £5,935)** |
|  |  |  |  |  |
| Collagenase |  |  |  |  |
|  | Number of participants who received collagenase (N=332) | | | |
| Staff cost (Injection) | 40 | Minute | £143 (£51) | (£64 to £395) |
| Collagenase cost | 1 (0.04) | Injection | £581 (£25) | (£572 to £650) |
| Additional meds | 0 (1) | Item | £0 (£1) | (£0 to £9) |
| Total collagenase administration |  |  | **£725 (£58)** | **(£636 to £967)** |
| Staff cost (Manipulation) | 50 (15) | Minute | £167 (£50) | (£38 to £342) |
| Anaesthetic (Manipulation) | 0.9 (0.2) | Session | £116 (£30) | (£0 to £124) |
| Total joint manipulation |  |  | **£283 (£59)** | **(£95 to £466)** |
| Total collagenase cost |  |  | **£1,008 (£94)** | **(£743 to £1,433)** |

Table 4: Cost of healthcare resource use

| 3 months | LF group (N=260)  Mean (SD) | Collagenase group (N=289)  Mean (SD) | Mean difference (95% CI) |
| --- | --- | --- | --- |
| Total primary care | £42.6 (£128.4) | £15.6 (£57.6) | -£27.0 (-£43.4 to -£10.6) |
| Total secondary care | £255.0 (£473.4) | £216.0 (£822.3) | -£39.0 (-£153.2 to £75.1) |
| Medication | £15.1 (£20.8) | £16.6 (£22.3) | £1.5 (-£2.1 to £5.2) |
| Total healthcare resource use cost^a^ | £312.7 (£502.2) | £248.2 (£831.8) | -£64.5 (-£181.2 to £52.3) |
| 6 months | LF group (N=251) | Collagenase group (N=284) | Mean difference (95% CI) |
| Total primary care | £10.7 (£65.2) | £7.9 (£44.6) | -£2.8 (-£12.2 to £6.6) |
| Total outpatient visit | £24.3 (£88.7) | £31.2 (£202.3) | £6.9 (-£20.2 to £34.0) |
| Total secondary care | £70.8 (£444.5) | £124.0 (£718.5) | £53.2 (-£51.2 to £157.5) |
| Medication | £15.7 (£20.3) | £15.1 (£21.3) | -£0.6 (-£4.2 to £2.9) |
| Total healthcare resource use cost^a^ | £97.3 (£452.8) | £147.0 (£736.4) | £49.8 (-£55.7 to £155.2) |
| 1 year | LF group (N=258) | Collagenase group (N=293) | Mean difference (95% CI) |
| Total primary care | £7.6 (£49.5) | £3.0 (£17.8) | -£4.6 (-£10.7 to £1.5) |
| Total secondary care | £93.0 (£515.4) | £217.2 (£1,025.3) | £124.2 (-£14.2 to £262.9) |
| Medication | £14.4 (£21.2) | £16.1 (£23.3) | £1.8 (-£2.0 to £5.5) |
| Total healthcare resource use cost^a^ | £114.9 (£518.8) | £236.3 (£1,029.2) | £121.4 (-£17.8 to £260.6) |
| 2 years | LF group (N=209) | Collagenase group (N=239) | Mean difference (95% CI) |
| Total primary care | £1.0 (£8.0) | £5.4 (£38.2) | £4.4 (-£0.9 to £9.7) |
| Total secondary care | £410.2 (£1,881.2) | £240.9 (£703.3) | -£169.2 (-£426.7 to £88.3) |
| Medication | £16.0 (£21.8) | £19.4 (£23.7) | £3.5 (-£0.8 to £7.7) |
| Total healthcare resource use cost^a^ | £427.1 (£1,884.8) | £265.7 (£707.4) | -£161.3 (-£419.5 to £96.8) |
| ^a^ Including healthcare resource use both related and unrelated to hand conditions. | | | |

**Table 5: Summary of EQ-5D utility scores at each time point (all available cases)**

|  | LF group | | Collagenase group | | Mean difference (95% CI) |
| --- | --- | --- | --- | --- | --- |
| Time point | **Available cases** | **Mean (SD)** | **Available cases** | **Mean (SD)** |  |
| Baseline | 329 | 0.794 (0.170) | 332 | 0.791 (0.174) | -0.003 (-0.029 to 0.024) |
| 2 weeks | 253 | 0.715 (0.146) | 281 | 0.776 (0.139) | 0.061 (0.037 to 0.085) |
| 6 weeks | 234 | 0.792 (0.138) | 275 | 0.822 (0.144) | 0.03 (0.005 to 0.055) |
| 3 months | 249 | 0.859 (0.131) | 278 | 0.848 (0.168) | -0.011 (-0.037 to 0.015) |
| 6 months | 243 | 0.871 (0.149) | 264 | 0.858 (0.159) | -0.013 (-0.040 to 0.014) |
| 1 year | 247 | 0.865 (0.158) | 281 | 0.839 (0.178) | -0.026 (-0.055 to 0.003) |
| 2 years | 196 | 0.861 (0.166) | 227 | 0.817 (0.183) | -0.044 (-0.077 to -0.010) |

Figure 1 Distributions of costs and QALYs for LF and collagenase at 1-year and 2-year follow-ups

Figure 2 Threshold analysis of price of collagenase

References

1. Curtis LA, Burns A. Unit Costs of Health & Social Care 2020. Unit Costs of Health and Social Care. Kent: PSSRU, University of Kent; 2020.

2. 2019/20 National Cost Collection Data Publication [Internet]. 2021 [cited 10 May 2022]. Available from: <https://www.england.nhs.uk/publication/2019-20-national-cost-collection-data-publication/>.

3. Mehta S, Belcher HJ. A single-centre cost comparison analysis of collagenase injection versus surgical fasciectomy for Dupuytren's contracture of the hand. J Plast Reconstr Aesthet Surg. 2014;67(3):368-72.

4. Hospital Admitted Patient Care Activity 2019-20 [Internet]. 2020 [cited 10 May 2022]. Available from: <https://digital.nhs.uk/data-and-information/publications/statistical/hospital-admitted-patient-care-activity/2019-20>.

5. Prescription Cost Analysis – England 2020/21 [Internet]. 2021 [cited 10 May 2022]. Available from: <https://www.nhsbsa.nhs.uk/statistical-collections/prescription-cost-analysis-england/prescription-cost-analysis-england-202021>.

6. BNF. BNF 74 (British National Formulary) September 2017. London: BMJ Group and Pharmaceutical Press; 2018.

7. BNF. BNF 76 (British National Formulary) September 2018 2018 [

8. Dias J, Braybrooke J. Dupuytren's contracture: an audit of the outcomes of surgery. The Journal of Hand Surgery: British and European Volume. 2006;31(5):514-21.

9. Dias J, Singh H, Ullah A, Bhowal B, Thompson J. Patterns of recontracture after surgical correction of Dupuytrens disease. Journal of Hand Surgery (American Volume). 2013;38:1987-93.

10. Peimer C, Blazar P, Coleman S, Kaplan F, Smith T, Tursi J, et al. Dupuytren contracture recurrence following treatment with collagenase clostridium histolyticum (CORDLESS study): 3-year data. The Journal of hand surgery. 2013;38(1):12-22.

11. Peimer C, Blazar P, Coleman S, Kaplan F, Smith T, Lindau T. Dupuytren contracture recurrence following treatment with collagenase clostridium histolyticum (CORDLESS [Collagenase Option for Reduction of Dupuytren Long-Term Evaluation of Safety Study]): 5-year data. The Journal of hand surgery. 2015;40(8):1597-605.

12. van Rijssen AL, ter Linden H, Werker PMN. Five-Year Results of a Randomized Clinical Trial on Treatment in Dupuytren's Disease: Percutaneous Needle Fasciotomy versus Limited Fasciectomy. Plastic and Reconstructive Surgery. 2012;129(2).

13. Brazzelli M, Cruickshank M, Tassie E, McNamee P, Robertson C, Elders A, et al. Collagenase clostridium histolyticum for the treatment of Dupuytren’s contracture: systematic review and economic evaluation. Health Technology Assessment. 2015.

14. Office for national statistics (ONS). Deaths registered in England and Wales: 2020 edition 2020 [Available from: <https://www.ons.gov.uk/peoplepopulationandcommunity/birthsdeathsandmarriages/deaths/datasets/deathsregisteredinenglandandwalesseriesdrreferencetables>.
